# Determination of Zearalenone and Trichothecenes, Including Deoxynivalenol and Its Acetylated Derivatives, Nivalenol, T-2 and HT-2 Toxins, in Wheat and Wheat Products by LC-MS/MS: A Collaborative Study

**DOI:** 10.3390/toxins12120786

**Published:** 2020-12-10

**Authors:** Annalisa De Girolamo, Biancamaria Ciasca, Michelangelo Pascale, Veronica M.T. Lattanzio

**Affiliations:** Institute of Sciences of Food Production, National Research Council of Italy, via Amendola 122/O, 70126 Bari, Italy; annalisa.degirolamo@ispa.cnr.it (A.D.G.); biancamaria.ciasca@ispa.cnr.it (B.C.); michelangelo.pascale@ispa.cnr.it (M.P.)

**Keywords:** trichothecenes, zearalenone, *Fusarium* toxins, wheat, liquid chromatography–mass spectrometry, official control, collaborative study

## Abstract

An analytical method for the simultaneous determination of trichothecenes—namely, nivalenol (NIV), deoxynivalenol (DON) and its acetylated derivatives (3- and 15-acetyl-DON), T-2 and HT-2 toxins—and zearalenone (ZEN) in wheat, wheat flour, and wheat crackers was validated through a collaborative study involving 15 participants from 10 countries. The validation study, performed within the M/520 standardization mandate of the European Commission, was carried out according to the IUPAC (International Union of Pure and Applied Chemistry) International Harmonized Protocol. The method was based on mycotoxin extraction from the homogenized sample material with a mixture of acetonitrile-water followed by purification and concentration on a solid phase extraction column. High-performance liquid chromatography coupled with tandem mass spectrometry was used for mycotoxin detection, using isotopically labelled mycotoxins as internal standards. The tested contamination ranges were from 27.7 to 378 μg/kg for NIV, from 234 to 2420 μg/kg for DON, from 18.5 to 137 μg/kg for 3-acetyl-DON, from 11.4 to 142 μg/kg for 15-acetyl-DON, from 2.1 to 37.6 μg/kg for T-2 toxin, from 6.6 to 134 μg/kg for HT-2 toxin, and from 31.6 to 230 μg/kg for ZEN. Recoveries were in the range 71–97% with the lowest values for NIV, the most polar mycotoxin. The relative standard deviation for repeatability (RSD_r_) was in the range of 2.2–34%, while the relative standard deviation for reproducibility (RSD_R_) was between 6.4% and 45%. The HorRat values ranged from 0.4 to 2.0. The results of the collaborative study showed that the candidate method is fit for the purpose of enforcing the legislative limits of the major *Fusarium* toxins in wheat and wheat-based products.

## 1. Introduction

The mycotoxins nivalenol (NIV); deoxynivalenol (DON) and its acetyl derivatives, 3-acetyl DON (3-AcDON) and 15-acetyl DON (15-AcDON); T-2 toxin (T-2) and its metabolite HT-2 toxin (HT-2); and zearalenone (ZEN) are produced by various *Fusarium* species. Cereals such as wheat, maize, barley, oats, rye, and relevant derived products are most likely to be affected [1]. The toxic effects of *Fusarium* toxins on human and animal species have been extensively studied [2,3,4,5]. Because of its ability to induce acute vomiting in pigs, DON has also been given the trivial name “vomitoxin”. Its acute effects in humans are similar to those observed in animals. The most common effects of long-term dietary exposure to DON are weight gain suppression and anorexia [2]. Similarly, toxicity studies have shown that NIV has anorectic effects upon short-term exposure, as well as immunotoxic and hematotoxic effects [5]. T-2 and HT-2 are known to inhibit the synthesis of protein, DNA, and RNA, and to have immunosuppressive and cytotoxic effects [4]. Estrogenic activity is the critical mode of action of ZEN and its main reductive metabolites. Adverse effects have been reported on the reproductive tract either in male and female animals, including testosterone synthesis, spermatogenesis, fertility, and embryo survival [3]. According to the available toxicological data concerning carcinogenicity in humans, ZEN, DON, and NIV were included by the World Health Organization’s (WHO) International Agency for Research on Cancer (IARC) in the Group 3 as not classifiable regarding their carcinogenicity to humans [6].

To ensure the safety of cereals and derived products, the European Commission (EC) set maximum levels for mycotoxins, including DON and ZEN [7]. Regarding T-2 and HT-2, indicative levels to stimulate data collection for the sum of these *Fusarium* mycotoxins in cereals and cereal-derived products were published in the Commission Recommendation 2013/165/EU [8]. When evaluating the risks to human and animal health related to the presence of DON, its acetylated derivatives (3- and 15-AcDON) and the modified form DON-3-glucoside, as well as T-2 and HT-2 in food and feed, the European Food Safety Authority (EFSA) recommended the interlaboratory validation and standardization of LC–MS/MS methodologies for the simultaneous quantification of DON and its derivatives and analytical methods with an appropriate sensitivity for T-2 and HT-2 toxins in food and feed commodities. In addition, the collection of analytical data on the co-occurrence of the above-mentioned toxins was requested [2,9]. Even though not regulated yet, available studies on the occurrence and toxicity of NIV have been evaluated by the EFSA, highlighting the need for validated methods suitable for the determination of NIV in the low μg/kg range to provide reliable data for risk assessment [5,10].

The establishment of standardized methods of analysis is of the utmost importance to guarantee a uniform application of the European legislation in all member states and contribute to maintaining a high level of food and feed safety. The results of collaborative studies and proficiency tests (PTs) made available in the last decade have provided evidence that liquid chromatography-mass spectrometry (LC-MS)-based methods of analysis for (multiple) mycotoxins in food/feed can be actually fit for the purpose of the enforcement of legislative limits [11,12,13]. At the EU level, standardized methods of analysis are established by the European Committee for Standardization (CEN). The first standard LC-MS-based methodologies for the determination of mycotoxins in foods were published in 2017 by the CEN and cover ZEN [14] and T-2/HT-2 toxins [15] determination. These two methods were validated within the M/520 standardization mandate [16] by which the Commission invited CEN to establish European Standards/Technical Specifications that provide standardized methods of analysis for mycotoxins in food. Six of the 11 methods of analysis listed in this mandate were specifically requested to be based on LC-MS.

The work described herein addresses item 4 of the standardization mandate, aiming at optimizing and validating an analytical method for the simultaneous determination of NIV, DON and its acetyl derivatives (3-AcDON and 15-AcDON), T-2, HT-2, and ZEN in cereals and cereal products by liquid chromatography-tandem mass spectrometry (LC-MS/MS). According to the EC sector information and statistics on cereals (https://ec.europa.eu/info/food-farming-fisheries/plants-and-plant-products/plant-products/cereals_en), more than 50% of the cereals grown in the EU are wheat. It was therefore agreed within the working group (CEN TC275/WG5 biotoxins) to select wheat and wheat products as representative target commodities for the validation study. The results of the full collaborative study, involving seventeen international laboratories, are reported together with information relevant to the preparation and characterization of the test materials, the protocol of analysis, and the statistical analysis of the results. The derived method has been recently adopted as a CEN standard [17].

## 2. Results and Discussion

### 2.1. Pre-Trial Results

Prior to the full validation study, laboratories had to participate in a pre-trial study. The aims of the pre-trial were to let the participants become familiarized with the correct execution of the method protocol and to optimize the LC-MS/MS conditions for mycotoxin detection, as well as to pre-check the method interlaboratory performances.

The results forms were returned by twelve out of fifteen laboratories participating in the pre-trial. One laboratory was removed because of the extremely low values reported for all target mycotoxins. A root cause analysis performed in collaboration with the participant laboratory lead us to identify a possible problem in the extract clean-up, leading to very high matrix effects which could not be properly compensated by the internal standards. Two laboratories reporting valid results were excluded from the statistical evaluations for 3-AcDON and 15-AcDON since they reported values as the sum of 3-AcDON and 15-AcDON.

The recoveries from spiked wheat flour were in the range of 78–102%, with relative standard deviations for repeatability (RSD_r_) ranging from 4.4% to 12% and relative standard deviations for reproducibility (RSD_R_) ranging from 8.9% to 25%. For contaminated wheat samples, RSD_r_ and RSD_R_ ranged from 3.2% to 17% and from 7.5% to 29%, respectively.

The Horwitz ratio (HorRat) values, calculated as the ratio between the RSD_R_ obtained during the pre-trial study and the predicted RSD_R_ calculated by the modified Horwitz equation [18,19], were between 0.5 and 2.1 for all mycotoxins in the three test materials. In all the considered cases, the method performances fell within the criteria established by the European Commission for the acceptability of methods for mycotoxin determination, set for DON, ZEN, and T-2/HT-2 [20,21], proving the candidate method to be suitable for the full collaborative study.

**Figure 1:**
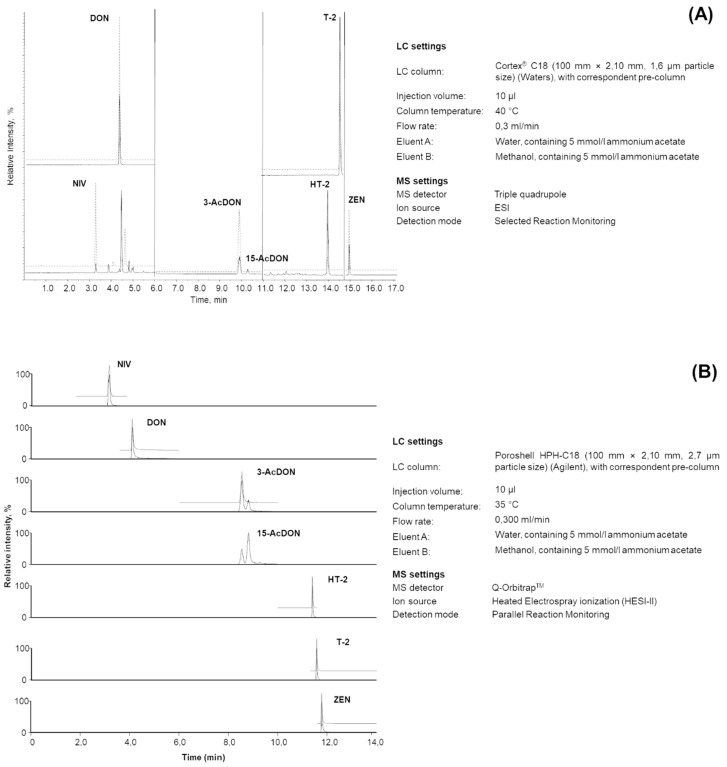
(**A**) Selected ion chromatogram (quantifier SRM–selected ion monitoring-transitions) and LC-MS settings of a wheat sample spiked with 120 µg/kg nivalenol (NIV), 600 µg/kg deoxynivalenol (DON), 75 µg/kg 3- and 15-AcDON (acetyl deoxynivalenol), 25 µg/kg HT-2 toxin (HT-2) and T-2 toxin (T-2), 50 µg/kg zearalenone (ZEN), and relevant isotopically labeled internal standard (ISTD—upper lines); (**B**) extracted ion chromatogram (quantifier ions) and LC-MS settings of a mycotoxin standard solution containing 1.87 µg/mL NIV, 9.37 µg/mL DON, 1.12 µg/mL 3- and 15-AcDON, 0.37 µg/mL HT-2 and T-2, 0.75 µg/mL ZEN, and relevant ISTD (upper lines).

Some issues related to the method protocol execution arose and were discussed with the participants. Two participant laboratories were not able to achieve a full chromatographic separation of 3-AcDON and 15-AcDON. Two participants reporting poor peak shapes for NIV were identified as outliers by the Cochran test. Therefore, improvements of the LC conditions were suggested by the interlaboratory study coordinator and examples of suitable LC-MS/MS settings were provided (Figure 1A,B).

### 2.2. Full Collaborative Study

Seventeen laboratories participated in the full collaborative study, and 15 report forms were collected. Eleven participants used triple quadrupole mass detectors (in a wide range of manufacturers and models), whereas four used hybrid quadrupole-Orbitrap^TM^ detectors. Given the limited number of participants using high-resolution MS (HRMS) detectors, low- and high-resolution MS data were evaluated as a unique set.

Three participants were not able to resolve 3- and 15-AcDON peaks and reported results relevant to the sum of the two isomers. Two participants could report data for 3-AcDON only, since 3-AcDON and 15-AcDON were detected in negative and positive ion mode, respectively, in two separate chromatographic runs, and it was not possible to quantitate 15-AcDON with the response ratio of the isotopically labelled 3-AcDON. Moreover, two participants using a triple quadrupole MS detector with polarity switching between defined retention time windows did not detect ZEN. This was probably due to slight retention time shifts leading ZEN to elute out of the dedicated retention time period.

Some data were considered “invalid” and were excluded from the statistical evaluation. Specifically, one laboratory reported problems in chromatography (poor peak shape) and provided extremely high values for the majority of the data. One laboratory using HRMS reported extremely high values in all samples for early eluting toxins (NIV and DON), probably due to poor separation from the solvent front. One participant faced sensitivity issues in all the samples but calibrants and reported extremely low values for the majority of the toxin/sample combinations. The cause was not clearly identified, but it was reasonably attributed to problems occurring during the clean-up procedure.

The results of the remaining laboratories were subjected to statistical analysis for the identification of outliers by the Cochran and Grubbs test to remove laboratories showing a significantly higher variability among replicates and extreme values, respectively [22]. For all toxin/material combinations, the number of identified outliers was lower than 20%, and thus below the maximum of 2/9 laboratories that could be removed as recommended in the AOAC (Association of Official Analytical Chemists) International guidelines for conducting interlaboratory studies [22].

Full collaborative study results are reported in Table 1. The method was validated with the perspective to be applied for compliance testing by official food control laboratories. Therefore, it was aimed at achieving method performance characteristics to meet the provisions in the Commission Regulation No. 401/2006/EC and Commission Recommendation 519/2014/EU [20,21]. The mycotoxin levels in contaminated materials were set in order to encompass legal limits (were available), which were also chosen as spiking levels for recovery assessment (Table 1).

The recoveries were in the range of 71–97%, and the lowest values (71–78%) were obtained for NIV, the most polar mycotoxin. The relative standard deviations for repeatability (RSD_r_) were in the range of 2.2–34%, and the relative standard deviations for reproducibility (RSD_R_) were between 6.4% and 45%. Almost all the data fulfilled the EC acceptability criteria [20,21]. In a few cases, RSD_R_ values higher than 40% were obtained—namely, for 3-AcDON at 75 µg/kg (wheat flour), 15-AcDON at 36 µg/kg (wheat A), 24 µg/kg (wheat flour B), 11 µg/kg (wheat crackers A), and for T-2 at 48 µg/kg (wheat flour B). Overall, the worst precision values were obtained for 15-AcDON. This could be attributed either to the lower number of valid results or the use of ^13^C_17_-3-AcDON as an internal standard.

**Figure 2:**
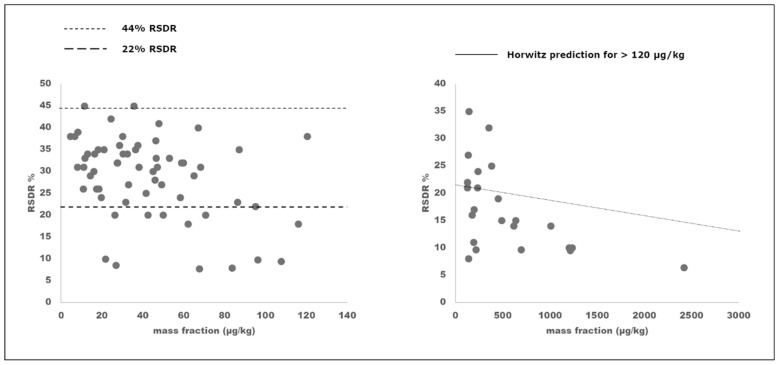
Reproducibility data obtained for all trichothecenes and ZEN in wheat, wheat flour, and cracker test materials in the mass fraction ranges 0–120 µg/kg and 120–2500 µg/kg.

The expected HorRat values, based on historical performance data and used as a guide to determine the acceptability of the precision of a method, are between 0.5 and 1.5, although the limits for performance acceptability are 0.5–2.0 [22]. In this study, the HorRat values ranged from 0.4 to 2.0, being in some cases slightly better than the expected ones on the basis of historical data (Table 1 and Figure 2). The performances of this collaborative study are in line with the data reported for other very recent collaborative studies for the validation of multimycotoxin LC-MS/MS methods [13,23], reporting HorRat values down to 0.2. Besides the expertise of the participating laboratories, the improved performances of the LC-MS/MS multimycotoxin methods can be attributed to the use of isotopically labelled internal standards, which improved the method precision by compensating for LC-MS signal drift over long analysis sequences as well as for matrix effects.

Overall, the method validation results indicated that the focused mycotoxins can be reliably detected at levels encompassing the legal limits.

Figure 2 depicts plots of RSD_R_ vs. mass fraction in the range 0–120 µg/kg (a) and 120–2500 µg/kg (b). The RSD_R_ values were mostly between 22% and 45%.

Looking at the overall results, the main factors influencing recoveries and precision turned out to be the chemical nature of the toxin. Lower but still acceptable recoveries (≥71%) were obtained for NIV. With this being the most polar toxin targeted by the method, some analyte losses (higher than other mycotoxins) could occur in the solid phase extraction (SPE) clean-up loading/washing steps. Furthermore, a good chromatographic separation between 3- and 15-AcDON was confirmed to be critical to achieve satisfactory method performance results for both isomers. On the other hand, no clear influence of the matrix was observed on recoveries and precision, suggesting that the method can be considered “horizontal”—i.e., applicable to different commodities without changing/adapting the sample preparation and analysis procedure.

## 3. Conclusions

A method for the simultaneous determination of zearalenone and the major trichothecenes—namely, nivalenol, deoxynivalenol and its acetyl derivatives (3-acetyl DON and 15-acetyl DON), and HT2-and T-2 toxins—was validated in an international collaborative study involving 15 participant laboratories from 10 countries according to the AOAC/IUPAC (International Union of Pure and Applied Chemistry) International Harmonized Protocol [22]. The method was based on LC-MS/MS detection after SPE extract clean-up. The results of the collaborative study met the required method performance criteria, with HorRat values in the range of 0.4–2.0. Data from the collaborative study as well as feedback from participants confirmed that the chromatographic separation of 3-AcDON and 15-AcDON remains one of the challenging steps of the method, as well as achieving satisfactory recoveries for NIV, which were higher than 70%. Even though affecting the cost-effectiveness of the method, the use of isotopically labeled internal standards is essential to manage the matrix effect, contributing to achieve a satisfactory precision. Finally, the applied generic sample preparation procedure, based on polymeric SPE columns, has been already demonstrated to be applicable to a wide range of mycotoxins and food commodities [24], suggesting possible further extensions of the method scope. The method complies with the provisions of the commission regulations setting performance criteria for mycotoxin methods [20,21] and has been recently adopted as a CEN standard [17].

## 4. Materials and Methods

### 4.1. Chemicals and Reagents

Participants in the collaborative study were requested to use only reagents of recognized analytical grade and specifically the following: water, acetonitrile, and methanol of HPLC quality; ammonium acetate for mass spectrometry, c(CH_3_COONH_4_) ≥ 99.0%; solid-phase extraction columns Oasis^®^ HLB 3 mL, 60 mg (Waters, Milan, Italy), or equivalent; standard NIV, DON, 3Ac-DON, 15Ac-DON, HT-2, T-2, ZEN crystalline, as a film or as certified standard solution; isotopically labelled internal standards (^13^C_15_-NIV, ^13^C_15_-DON, ^13^C_17_-3-AcDON, ^13^C_22_-HT-2, ^13^C_24_-T-2, ^13^C_18_-ZEN) as acetonitrile solutions (25 μg/mL).

### 4.2. Test Materials

Three commodities—namely, common wheat (cereal), wheat flour (cereal flour), and wheat crackers (cereal-based food)—were selected. For each food commodity, one blank (containing mycotoxins at levels below the detection limit of the in-house validated method—i.e., 10 µg/kg NIV; 5 µg/kg DON; 1 µg/kg 3-AcDON and 15-AcDON; 0.5 µg/kg HT-2, T-2, and ZEN), one blank for spiking purposes (for recovery evaluation) and three contaminated materials (low, medium, and high level) with target mycotoxins were prepared. Desired mycotoxin levels in contaminated test materials were within a contamination range encompassing the EU maximum permitted levels (where applicable) (Table 1). All the materials were dispensed in plastic bottles (approx. 30 g each container) that were labeled, sealed, and stored at −20 °C until analysis for homogeneity and stability study or dispatch for collaborative trial testing.

#### 4.2.1. Preparation of Whole Soft Wheat and Soft Wheat Flour Test Materials

Uncontaminated common wheat kernels and soft wheat flour (about 10 kg each) were selected among a set of samples purchased from the Italian retail market. The absence of contamination was verified by analysis for the mycotoxin content according to relevant validated/reference methods [25,26,27]. Wheat kernels were milled by an ultra-centrifugal mill (ZM 200, Retsch, 500 μm sieve). About 4 kg of each commodity were used as blank, whereas the remaining 6 kg were used for the preparation of the contaminated materials.

Since naturally contaminated materials containing all focused mycotoxins at the desired levels were unavailable at the time of this study, the preparation of contaminated test materials was performed according to a previously developed protocol [12]. Briefly, contaminated test materials were prepared by mixing and homogenizing blank material (common wheat or common wheat flour) with culture extracts of *Fusarium* toxigenic species (deposited at the Institute of Sciences of Food Production collection, http://www.ispa.cnr.it/Collection) grown on wheat kernels. Fungal cultures of *F. graminearum* ITEM 126 (producing DON, ZEN, and 3-AcDON) and *F. sporotrichioides* ITEM 707 (producing T-2 and HT-2) were dried, ground, extracted, and analyzed for the mycotoxin content according to relevant validated/reference methods [25,26,27]. Subsequently, a multi-mycotoxin spiking solution containing NIV, DON, 3- and 15-AcDON, T-2, HT-2, and ZEN was prepared by mixing adequate amounts of culture extracts and standard mycotoxin solutions (for NIV and 15-AcDON). For each contamination level (low, medium, high), 2 kg aliquots of blank material were split into 0.5 kg portions and fortified with the multi-mycotoxin solution. After solvent evaporation (overnight at room temperature), the 0.5 kg portions were pooled and passed through an ultra-centrifugal mill (ZM 200, Retsch, 500 μm sieve), then homogenized by a laboratory mixer for 24 h.

This procedure resulted in three contaminated wheat materials (low, medium, and high level) and three contaminated wheat flour materials (Table 1).

#### 4.2.2. Preparation of Wheat Crackers Test Materials

Wheat crackers were prepared at the laboratory scale according to the following optimized recipe: 500 g of common wheat flour, 40 g sunflower seed, 200 mL of water, 10 g of salt. Ingredients were mixed up in a kneading machine (Princess 151 936 Silver, Milan, Italy) for 20 min. The dough was rolled out with a traditional pasta machine (model 150 Atlas Marcato SpA, Italy) into sheets of 2 mm, cut, and baked in a conventional oven at 230 °C for 10 min. Mycotoxin-contaminated wheat crackers were prepared by adding the appropriate amount of mycotoxin standard solutions to the water necessary for dough preparation. After baking, each material (blank or contaminated) was passed through an ultra-centrifugal mill (ZM 200, Retsch, 500 μm sieve), then homogenized by a laboratory mixer for 24 h.

#### 4.2.3. Homogeneity of Test Materials

A homogeneity study was carried out according to the procedure described by ISO guide 35:2006 [28] on randomly selected units: specifically, 16 units (for blank test materials) and 11 units (for contaminated test materials) of about 25 g were taken at systematic intervals from the filling sequence. Each unit of 25 g was divided into two aliquots and analyzed in duplicate under repeatability conditions. The analytical method used for homogeneity testing was the one described in this protocol, keeping the same ratio of test portion to extraction solvent.

Homogeneity was statistically evaluated according to ISO 13528:2015 [29] and F-test. The parameters considered for the homogeneity test were the analytical precision (within bottle standard deviation, *s_w_*–analytical SD) and the heterogeneity standard deviation (between bottle standard deviation, s_b_–heterogeneity SD). The F-test was used to determine whether the observed s_b_ deviated significantly from the *s_w_*.

The heterogeneity (*s_b_*) was then compared to the target standard deviation (*s*). The *s* values were obtained using the truncated Horwitz equation corrected by Thompson—i.e., if the relative target standard deviation according to Horwitz was greater than 22%, it was truncated to 22%. The samples were considered to be adequately homogenous if s_b_ ≤0.3 s [28,29]. Data were processed using the ProLab software (ProLab Software—QuoData, Drezden—www.quodata.de).

All the test materials passed the homogeneity test and turned out to be appropriate for the collaborative study.

#### 4.2.4. Stability of Test Materials

Randomly selected units of the test materials were submitted to accelerated ageing at different temperatures (4 °C, 20 °C, and 60 °C) over a total period of 1.5 months according to the so-called isochronous short-term stability study [28,29,30]. A total of 26 bottles for each material were stored at −20 °C (reference temperature), then 2 random bottles per time were moved to the different temperatures after 0.25, 0.50, 1 and 1.5 month for a total of 24 bottles. All the units were analyzed at the end of month 1.5, under repeatability conditions, together with 2 reference samples which were kept at −20 °C over the whole period of the short-term stability study. The analytical method used for stability testing was the one described in this protocol. The stability study was performed on each contamination level per material.

Statistical results assessment was performed according to ISO guide 35:2006 using the *t-*test to test the regression for significance [28,29]. Specifically, the evaluation of data was carried out by performing a linear regression on the experimentally determined concentrations of each mycotoxin (mean values) versus time (days). For a stable material, it is expected that the intercept is equal to the reference value, whereas the slope does not differ significantly from zero.

The evaluation of data from the short-term stability study indicated that no significant trend was observed for the test samples at all temperature conditions (4 °C, 20 °C, and 60 °C) for the time span of the collaborative study. It was concluded that the three test materials were stable for at least 1.5 months following their preparation.

### 4.3. Collaborative Study

#### 4.3.1. Study Layout

Prior to the full validation study, laboratories had to participate in a pre-trial study to let them become familiarized with the correct execution of the method protocol and optimize the LC-MS/MS conditions for mycotoxin detection. To this scope, they received:

- One blank wheat flour sample, to be used for five determinations (two determinations as blank and three determinations for recovery check).
- One wheat sample (to be analyzed as blind duplicate) contaminated with 1298 µg/kg DON; 58 µg/kg HT-2; 8.3 µg/kg T-2; 148 µg/kg ZEN.
- A mixed stock solution (stock solution B, see Section 4.3.2) and a mixed standard solution in acetonitrile to be used for spiking purposes and calibrants preparation, respectively, and a mixed isotopically labeled internal standard (ISTD) solution in acetonitrile (mixed ISTD, see Section 4.3.2) to be used as internal standard.
- Columns for solid phase extraction (SPE) clean-up.
- Method protocol in SOP (Standard Operating Procedure) format and reporting sheets.

The full validation study was planned according to the requirements of the IUPAC/AOAC international harmonized protocol [22,31]. The main purpose of the collaborative study was to estimate the precision of the candidate method under repeatability and reproducibility conditions. The method accuracy was evaluated by spiking experiments. Fifteen laboratories were involved in the trial, representing a cross-section of academia, official control, and private laboratories.

Participants received the following materials:

- Two blank materials per each commodity (wheat, wheat flour, wheat crackers), about 15 g each, to be used for two independent determinations as spiked sample for recovery checking. Participants were asked to fortify the material with the respective spiking solution with an evaporation time of approximately 1 h before the determination.
- Blind duplicates of 1 blank material and 3 contaminated materials (low, medium and high level see Table 1) per each commodity. Test material size (15 g) was sufficient to perform a single determination. Materials were coded in a random pattern.
- Three mixed mycotoxin stock solutions to spike the 3 target commodities respectively; a mixed standard solution for calibrant preparation; a mixed ISTD solution.
- Thirty (+ 2 extra) solid phase extraction (SPE) columns.
- Material receipt form.
- Standard operating protocol (SOP).
- Reporting sheets.

The results had to be expressed in micrograms per kilogram (µg/kg). Each laboratory was free to use its own LC-MS/MS set-up, and the optimization of settings for the LC and MS/MS detection was left to the participants. Mycotoxin detection was requested to be performed in Selected Reaction Monitoring (SRM) in case of MS/MS analyzers or Parallel Reaction Monitoring (PRM) in case of MS/high-resolution mass spectrometry (HRMS) analyzers. The full chromatographic separation of 3-AcDON and 15-AcDON was mandatory, and the participants were asked to provide individual data for the two toxins. The participants were requested to make available chromatograms for samples and calibration standards and to provide the following details on the applied LC-MS/MS instrumentation and method settings: LC column characteristics, mobile phase composition and gradient elution, flow rate, injection volume, MS ion source, SRM or PRM ions.

#### 4.3.2. Mycotoxin Solutions

The following mixed mycotoxin solutions were prepared in acetonitrile, according to concentrations specified in the following:

- Mixed stock solution A, to be used for wheat spiking: NIV, 12.5 μg/mL; DON, 62.5 μg/mL; 3-AcDON, 7.5 μg/mL; 15-AcDON, 7.5 μg/mL; T-2, 2.5, μg/mL; HT-2, 2.5 μg/mL; ZEA, 5.0 μg/mL.
- Mixed stock solution B, to be used for wheat flour spiking: NIV, 7.5 μg/mL; DON, 37.5 μg/mL; 3-AcDON, 3.75 μg/mL; 15-AcDON, 3.75 μg/mL; T-2, 1.25, μg/mL; HT-2, 1.25 μg/mL; ZEA, 3.75 μg/mL.
- Mixed stock solution C, to be used for wheat crackers spiking: NIV, 5 μg/mL; DON, 25 μg/mL; 3-AcDON, 2.5 μg/mL; 15-AcDON, 2.5 μg/mL; T-2, 0.625, μg/mL; HT-2, 0.625 μg/mL; ZEA, 2.5 μg/mL.
- Mixed standard solution, prepared by 10 times dilution with acetonitrile of the multi-toxin stock solution A. This solution was used to prepare calibrants (see Table 2).
- Mixed internal standard (ISTD) solution, prepared by mixing the commercial individual ISTD solutions to obtain a mixture containing ^13^C_15_-NIV, 1.25 µg/mL; ^13^C_15_-DON, 6.25 µg/mL; ^13^C_17_-3-AcDON, 0.75 µg/mL; ^13^C_22_-HT-2, 0.25 µg/mL; ^13^C_24_-T-2, 0.25 µg/mL; ^13^C_18_-ZEN, 0.5 µg/mL.

Aliquots of 0.5 mL of spiking solutions A, B, and C were dispensed in 2 mL amber vials. The spiking solutions were labeled as Vial #1, Vial #2, and Vial #3; mycotoxin concentrations were blind. Approximately 2 mL of mixed standard solution (labeled as Vial #4) and approximately 4 mL of mixed ISTD solution (labeled as Vial #5) were dispensed in 4 mL amber vials; the mycotoxin concentrations were specified.

To prepare the calibration solutions, different volumes of the mixed standard solution and the mixed ISTD solution were added to six autosampler vials as listed in Table 2 to obtain six calibration levels across the calibration range. After evaporation to dryness under a stream of air or nitrogen at approximately 40 °C, the dried residue was re-dissolved by adding 400 µL of HPLC injection solvent.

#### 4.3.3. Sample Preparation

The test samples (10 g) were extracted with 50 mL (V3) acetonitrile/water 84/16 (*v*/*v*) for 60 min on an orbital shaker. The extract was filtered through filter paper (Whatman No. 4), and 5 mL (V4) of filtrate (equivalent to 1 g sample) were evaporated to dryness at 40 °C under a stream of air. The residue was reconstituted with 100 µL of methanol and then 900 µL water were added (to obtain a methanol: water ratio of 10:90, *v*/*v*). The Oasis^®^ HLB column was activated and conditioned prior to use as follows. The column was attached to a vacuum manifold, conditioned with 2 mL methanol, and equilibrated with 2 mL methanol/water 10/90 (*v*/*v*). The reconstituted sample extract was then passed through the column at a flow rate of about one drop per second. The column was washed with 1 mL methanol/water 20/80 (*v*/*v*) and dried. Afterwards, the toxins were eluted with 1 mL methanol. To prepare the sample test solution, 100 µL of the mixed ISTD solution were added to the SPE eluate. Then the SPE eluate was evaporated to dryness under a stream of air or nitrogen at 40 °C. The dried residue was re-dissolved by adding 400 µL (V1) of HPLC injection solvent and filtered through 0.20 µm regenerated cellulose filter.

For the determination of the recoveries, spiking was performed with the mixed stock solutions A, B, and C for wheat, wheat flour, and wheat crackers, respectively, with an evaporation time of approximately one hour.

#### 4.3.4. LC-MS Analysis

The optimization of settings for LC and MS/MS detection was left to the participants. Examples of suitable LC-MS/MS settings were provided (they are reported in Figure 1A,B). Mandatory requirements were (i) the chromatographic separation of the two isomers 15-AcDON and 3-AcDON; (ii) using a tandem mass spectrometer (MS/MS), performing Selected Reaction Monitoring (SRM) in the case of MS/MS analyzers or Parallel Reaction Monitoring (PRM) in the case of MS/high-resolution mass spectrometry (HRMS) analyzers. For mycotoxin identification, it was requested to fulfill the criteria defined in the SANTE/12089/2016 document [32].

#### 4.3.5. Calculations

Mycotoxin quantification was performed by the isotopic dilution approach using 13C-fully labeled mycotoxins as internal standard. For each injection, the ratio of the peak area of each analyte to the peak area of the respective labelled analogue was calculated. The peak area of 15-AcDON was divided by the peak area of ^13^C_17_-3-AcDON. These peak area ratios are used in all subsequent calculations. A calibration curve for each of the seven analytes (NIV, DON, 3-AcDON, 15-AcDON, HT-2, T-2, and ZEN) was prepared by plotting the peak area ratios of each analyte calculated in the calibration solutions in Table 2 (*Y*-axis, dependent variable) against the corresponding amount (μg) of analyte injected on column (*X*-axis, independent variable). The mass fraction of each mycotoxin, w, in microgram per kilogram of the sample was calculated according to Formula (1):

(1) $$w\;=\;\left(\frac{R}{a}-\frac{b}{a}\right)\times \frac{V_{1}}{V_{2}}\times \frac{1000}{m_{SPE}}$$

where:

- *R* was the peak area ratio of the relevant analyte and the corresponding internal standard in the sample test solution;
- *a* was the slope of the calibration curve from calibration data, in µg^−1^;
- *b* was the intercept of the calibration curve from calibration data;
- *V*_1_ was the volume of the reconstituted extract after clean-up, here: 0.4 mL;
- *V*_2_ was the injection volume of the reconstituted sample extract, in milliliters;
- 1000 is a conversion factor;
- *m_SPE_* was the sample equivalent weight purified on SPE column, here: 1 g.

The sample equivalent weight (*m_SPE_*) was calculated according to Formula (2):

(2) $$m_{SPE}=\frac{m\;\times \;V_{4}}{V_{3}}$$

where:

- *m* was the mass of the extracted test portion, here: 10 g;
- *V*_3_ was the volume of the extraction mixture, here: 50 mL;
- *V*_4_ was the volume of filtered extract dried before clean-up, here: 5 mL.

## Figures and Tables

**Table 1:** Interlaboratory study results for trichothecenes and zearalenone in wheat, wheat flour, and wheat crackers. Spiking levels for recovery assessment were set to be equal to the EU maximum permitted (DON, ZEN) or recommended levels (HT-2 + T-2) where available.

	Material Description	No. of Labs ^a^	Mean (µg/kg)	Recovery (%)	RSD*_r_ (*%)	RSD*_R_*(%)	HorRat ^b^
NIV	Wheat, 250 µg/kg spike	14	194.1	78	5.8	17	0.8
	Wheat, contaminated A	12	87.12	n.a.	33	35	1.6
	Wheat, contaminated B	12	189.4	n.a.	11	11	0.6
	Wheat, contaminated C	13	377.8	n.a.	14	25	1.4
	Wheat flour, 150 µg/kg spike	11	116.0	77	6.1	18	0.8
	Wheat flour, contaminated A	14	52.89	n.a.	34	35	1.5
	Wheat flour, contaminated B	11	107.7	n.a.	4.2	9.4	0.4
	Wheat flour, contaminated C	11	214.8	n.a.	3.5	9.7	0.5
	Wheat crackers, 100 µg/kg spike	14	70.71	71	8.3	20	0.9
	Wheat crackers, contaminated A	12	27.69	n.a.	15	32	1.5
	Wheat crackers, contaminated B	13	64.87	n.a.	6.2	29	1.3
	Wheat crackers, contaminated C	13	123.8	n.a.	5.8	22	1.0
DON	Wheat, 1250 µg/kg spike	11	1212	97	3.7	9.5	0.6
	Wheat, contaminated A	12	635.4	n.a.	3.0	15	0.9
	Wheat, contaminated B	12	1201	n.a.	11	10	0.6
	Wheat, contaminated C	10	2420	n.a.	2.2	6.4	0.5
	Wheat flour, 750 µg/kg spike	11	692.5	92	5.0	9.7	0.6
	Wheat flour, contaminated A	14	351.0	n.a.	6.7	32	1.7
	Wheat flour, contaminated B	13	613.0	n.a.	3.8	14	0.8
	Wheat flour, contaminated C	11	1234	n.a.	3.3	10	0.7
	Wheat crackers, 500 µg/kg spike	13	448.7	90	2.8	19	1.0
	Wheat crackers, contaminated A	13	233.9	n.a.	14	24	1.2
	Wheat crackers, contaminated B	12	487.6	n.a.	5.0	15	0.8
	Wheat crackers, contaminated C	12	1005	n.a.	13	14	0.9
3AcDON	Wheat, 150 µg/kg spike	10	136.5	91	5.7	8.0	0.4
	Wheat, contaminated A	11	49.10	n.a.	3.0	27	1.2
	Wheat, contaminated B	10	83.68	n.a.	9.4	7.9	0.4
	Wheat, contaminated C	9	67.68	n.a.	6.4	7.7	0.4
	Wheat flour, 75 µg/kg spike	11	67.01	89	8.6	40	1.8
	Wheat flour, contaminated A	11	30.22	n.a.	6.9	34	1.5
	Wheat flour, contaminated B	11	58.95	n.a.	10	32	1.4
	Wheat flour, contaminated C	9	96.22	n.a.	5.3	9.8	0.4
	Wheat crackers, 50 µg/kg spike	13	44.96	90	5.9	30	1.4
	Wheat crackers, contaminated A	12	18.47	n.a.	17	26	1.2
	Wheat crackers, contaminated B	12	26.33	n.a.	6.7	20	0.9
	Wheat crackers, contaminated C	12	49.96	n.a.	12	20	0.9
15AcDON	Wheat, 150 µg/kg spike	11	141.8	95	8.5	35	1.6
	Wheat, contaminated A	8	35.64	n.a	5.8	45	2.0
	Wheat, contaminated B	8	41.48	n.a	9.3	25	1.1
	Wheat, contaminated C	8	30.14	n.a	7.1	38	1.7
	Wheat flour, 75 µg/kg spike	9	68.25	91	6.0	31	1.4
	Wheat flour, contaminated A	8	12.87	n.a	10	34	1.5
	Wheat flour, contaminated B	9	24.38	n.a	14	42	1.9
	Wheat flour, contaminated C	9	28.64	n.a	9.6	36	1.7
	Wheat crackers, 50 µg/kg spike	12	46.24	93	8.6	37	1.7
	Wheat crackers, contaminated A	9	11.41	n.a	25	45	2.0
	Wheat crackers, contaminated B	10	16.42	n.a	10	34	1.5
	Wheat crackers, contaminated C	11	32.34	n.a	5.9	34	1.5
HT-2	Wheat, 50 µg/kg spike	14	46.40	93	7.3	33	1.5
	Wheat, contaminated A	12	17.33	n.a.	6.6	26	1.2
	Wheat, contaminated B	14	36.29	n.a.	31	35	1.6
	Wheat, contaminated C	12	133.8	n.a.	10	27	1.2
	Wheat flour, 25 µg/kg spike	11	21.60	86	6.1	9.9	0.5
	Wheat flour, contaminated A	14	6.629	n.a.	26	38	1.7
	Wheat flour, contaminated B	13	14.18	n.a.	8.9	29	1.3
	Wheat flour, contaminated C	14	32.97	n.a.	9.2	27	1.2
	Wheat crackers, 12.5 µg/kg spike	12	10.73	86	4.7	26	1.2
	Wheat crackers, contaminated A	14	7.988	n.a.	14	31	1.4
	Wheat crackers, contaminated B	14	19.59	n.a.	15	24	1.1
	Wheat crackers, contaminated C	14	38.02	n.a.	11	31	1.4
T-2	Wheat, 50 µg/kg spike	12	45.89	92	12	28	1.3
	Wheat, contaminated A	12	27.50	n.a.	11	32	1.5
	Wheat, contaminated B	13	47.79	n.a.	34	41	1.9
	Wheat, contaminated C	12	18.01	n.a.	12	35	1.6
	Wheat flour, 25 µg/kg spike	13	20.85	83	6.3	35	1.6
	Wheat flour, contaminated A	13	11.53	n.a.	7.3	33	1.5
	Wheat flour, contaminated B	10	26.85	n.a.	6.3	8.5	0.4
	Wheat flour, contaminated C	13	37.57	n.a.	7.2	36	1.7
	Wheat crackers, 12.5 µg/kg spike	13	11.05	88	7.0	31	1.4
	Wheat crackers, contaminated A	13	4.530	n.a.	12	38	1.7
	Wheat crackers, contaminated B	14	8.091	n.a.	10	39	1.8
	Wheat crackers, contaminated C	13	15.88	n.a.	4.9	30	1.4
ZEN	Wheat, 100 µg/kg spike	10	95.12	95	6.5	22	1.0
	Wheat, contaminated A	11	59.85	n.a.	14	32	1.5
	Wheat, contaminated B	10	125.1	n.a.	12	21	1.0
	Wheat, contaminated C	10	229.7	n.a.	11	21	1.0
	Wheat flour, 75 µg/kg spike	11	62.03	83	13	18	0.8
	Wheat flour, contaminated A	11	42.49	n.a.	13	20	0.9
	Wheat flour, contaminated B	11	86.38	n.a.	24	23	1.1
	Wheat flour, contaminated C	11	171.7	n.a.	8.3	16	0.8
	Wheat crackers, 50 µg/kg spike	12	47.13	94	7.4	31	1.4
	Wheat crackers, contaminated A	10	31.62	n.a.	17	23	1.1
	Wheat crackers, contaminated B	9	58.26	n.a.	16	24	1.1
	Wheat crackers, contaminated C	11	120.4	n.a.	7.3	38	1.7

^a^ Number of laboratories (out of 15) remaining after the removal of invalid results and outliers. ^b^ HorRat (Horwitz Ratio) calculated using Predicted Standard Deviation from Thompson for concentrations >120 µg/kg and 22% for concentrations ≤120 µg/kg [19]. n.a.: not applicable.

**Table 2:** Preparation of the calibration solutions.

Mass Concentration of Calibration Solutions									
Calibration Solution	Mixed Standard Solution	Mixed ISTD Solution	NIV	DON	3-Ac DON	15-Ac DON	HT-2	T-2	ZEN
	µL	µL	µg/mL	µg/mL	µg/mL	µg/mL	µg/mL	µg/mL	µg/mL
1	25	100	0.078	0.391	0.047	0.047	0.016	0.016	0.031
2	50	100	0.156	0.781	0.094	0.094	0.031	0.031	0.063
3	100	100	0.313	1.563	0.188	0.188	0.063	0.063	0.125
4	200	100	0.625	3.125	0.375	0.375	0.125	0.125	0.250
5	400	100	1.250	6.250	0.750	0.750	0.250	0.250	0.500
6	600	100	1.875	9.375	1.125	1.125	0.375	0.375	0.750
**Mass Concentration of Isotopically Labelled Analytes (µg/mL) in All Calibration Solutions**									
			0.313	1.563	0.188	0.188	0.063	0.063	0.125
